# Gait performance and foot pressure distribution during wearable robot-assisted gait in elderly adults

**DOI:** 10.1186/s12984-017-0333-z

**Published:** 2017-11-28

**Authors:** Su-Hyun Lee, Hwang-Jae Lee, Won Hyuk Chang, Byung-Ok Choi, Jusuk Lee, Jeonghun Kim, Gyu-Ha Ryu, Yun-Hee Kim

**Affiliations:** 1 Department of Physical and Rehabilitation Medicine, Center for Prevention and Rehabilitation, Heart Vascular Stroke Institute, Samsung Medical Center, Sungkyunkwan University School of Medicine, Irwon-ro 81, Gangnam-gu, Seoul, 06351 Republic of Korea; 2 Department of Health Science and Technology, Department of Medical Device Management and Research, SAIHST, Sungkyunkwan University, Irwon-ro 81, Gangnam-gu, Seoul, 06351 Republic of Korea; 3 Department of Neurology, Neuroscience Center, Samsung Medical Center, Sungkyunkwan University School of Medicine, Irwon-ro 81, Gangnam-gu, Seoul, 06351 Republic of Korea; 4 Samsung Advanced Institute of Technology, Samsung Electronics, 130 Samsung-ro, Yeongtong-gu Gyeonggi-do, Suwon-si, 16678 Republic of Korea; 5 Office of Biomechanical science, Research Center for Future Medicine, Samsung Medical Center, Sungkyunkwan University, Irwon-ro 81, Gangnam-gu, Seoul, 06351 Republic of Korea

**Keywords:** Wearable hip-assist robot, Elderly adults, Gait, Spatiotemporal gait parameters, Muscle activity, Foot pressure distribution

## Abstract

**Background:**

A robotic exoskeleton device is an intelligent system designed to improve gait performance and quality of life for the wearer. Robotic technology has developed rapidly in recent years, and several robot-assisted gait devices were developed to enhance gait function and activities of daily living in elderly adults and patients with gait disorders. In this study, we investigated the effects of the Gait-enhancing Mechatronic System (GEMS), a new wearable robotic hip-assist device developed by Samsung Electronics Co, Ltd., Korea, on gait performance and foot pressure distribution in elderly adults.

**Methods:**

Thirty elderly adults who had no neurological or musculoskeletal abnormalities affecting gait participated in this study. A three-dimensional (3D) motion capture system, surface electromyography and the F-Scan system were used to collect data on spatiotemporal gait parameters, muscle activity and foot pressure distribution under three conditions: free gait without robot assistance (FG), robot-assisted gait with zero torque (RAG-Z) and robot-assisted gait (RAG).

**Results:**

We found increased gait speed, cadence, stride length and single support time in the RAG condition. Reduced rectus femoris and medial gastrocnemius muscle activity throughout the terminal stance phase and reduced effort of the medial gastrocnemius muscle throughout the pre-swing phase were also observed in the RAG condition. In addition, walking with the assistance of GEMS resulted in a significant increase in foot pressure distribution, specifically in maximum force and peak pressure of the total foot, medial masks, anterior masks and posterior masks.

**Conclusion:**

The results of the present study reveal that GEMS may present an alternative way of restoring age-related changes in gait such as gait instability with muscle weakness, reduced step force and lower foot pressure in elderly adults. In addition, GEMS improved gait performance by improving push-off power and walking speed and reducing muscle activity in the lower extremities.

**Trial registration:**

NCT02843828.

## Background

Robotic exoskeleton devices are intelligent systems designed for improvement of gait performance and activities of daily living (ADLs). Recently, several robot-assisted gait devices have been developed to enhance effective walking and to increase social interaction in elderly adults and people with gait disorders. Wearable robotics for rehabilitation is a developing field that is expected to grow as a solution to provide repetitive, high-dosage and high-intensity training [1]. Wearable robots could also be used to continue rehabilitation outside of a formal clinical setting, delivering intensive repetitive therapy at a reasonable cost [2, 3] and resolving challenges related to the design of portable therapy assistance devices [4].

In the past several decades, exoskeletons have undergone enormous progress and have been developed for many different applications all over the world. Exoskeletons can be classified into different types based on the part of the human body the exoskeleton supports (i.e. upper/lower extremity exoskeletons, full body exoskeletons, and specific joint support exoskeletons). With an ageing society and an increase in people with gait disorders, the use of lower extremity exoskeletons is promising for therapy assistance and gait rehabilitation. Some typical lower extremity exoskeletons have been developed and commercialized, according different applications and target users; the Robotic Orthosis Lokomat developed by Hocoma (Zurich, Switzerland) and Active Leg Exoskeleton (ALEX) developed by Banala et al. from the University of Delaware (Newark, DE, USA) for gait rehabilitation; the Ekso GT exoskeleton developed by Ekso Bionics (Richmond, CA, USA) for gait rehabilitation and human locomotion assistance; the Rewalk exoskeleton developed by ReWalk Robotics (Marlborough, MA, USA), the Vanderbilt exoskeleton developed by Goldfarb et al., and the CUHK-EXO developed at the Chinese University of Hong Kong for human locomotion assistance; the Hybrid Assistive Limb (HAL) developed at the University of Tsukuba in Tsukuba, Japan for human strength augmentation and gait rehabilitation; the Berkeley Lower Extremity Exoskeleton (BLEEX) and the Hanyang Exoskeleton Assistive Robot (HEXAR) developed by Hanyang University in Seoul, South Korea for Human strength augmentation [5]. In addition to the exoskeletons mentioned previously, many other lower extremity exoskeletons have been developed in all the world. To enhance walking performance and to increase the community mobility in elderly adults and patients with gait disorders, the Stride Management Assist (SMA®) System was developed by Honda R&D Corporation, Japan. The SMA® provides independent, active flexion and extension at each hip joint to assist the user during ambulation [2]. To help patients with mobility disorders, the LOPES (Lower-extremity Powered ExoSkeleton) was developed by the University of Twente (Enschede, The Netherlands) as a gait rehabilitation robot for treadmill training. To improve the physical abilities and strength of healthy people, the HERCULE exoskeleton was developed by RB3D in Auxerre, France. A soft lower extremity robotic exosuit was developed by Wehner et al., at Harvard University (Cambridge, MA, USA) to assist individuals with muscle weakness or patients who suffer from physical or neurological disorders [5].

Together with extended life expectancy, the rising elderly population experiences an increased incidence of pathologies (e.g., osteoarthritis, myopathies and hip pain) that affect walking ability by reducing muscle strength and endurance [6, 7]. Elderly adults with reduced muscle mass and weakened muscle strength may not be able to perform daily physical activities such as walking, and may also lose their stability during walking [5]. Age-related weakness and frailty related to sarcopenia affect quality of life (QOL) for elderly adults by decreasing the ability to perform many ADLs [8]. Physiological and anatomical changes in foot ligaments and bone lead to reduced step force, decreased stride length and increased variability in gait parameters. Furthermore, age is related to lower pressure under the hallux, midfoot and heel during gait. Overall, these age-related changes lead to decreases in gait and balance control ability and contribute to inefficient gait [9, 10]. Consequently, the elderly are at increased risk of experiencing one or more falls and their performance of ADLs and social participation is reduced.

With an ageing society, several rehabilitation interventions to improve age-related walking problems in older adults have been used in clinics. The multifactorial impairment-based therapeutic intervention aims to improve lower extremity strength, flexibility, and endurance capacities important for walking and involves therapeutic exercise to enhance capacities of muscle strength, range of motion, and aerobic conditioning. The task-oriented motor learning intervention aims to improve the motor skill of walking and may be a beneficial approach to improving walking in older adults [11]. Compared to these traditional rehabilitation interventions, exoskeleton-based rehabilitation has the advantages of releasing therapists from the heavy work of rehabilitation training, allowing intensive and highly repetitive training [5]. A powered exoskeleton robotic device that can reduce the muscle activity required to walk could help the elderly population recover normal walking and movement abilities [12].

Foot pressure analysis is clinically useful because it can prevent pressure ulcers in diabetics, guide the diagnosis of gait disorders and predict and reduce the risk of falling [13]. Even though many foot studies show extensive information on foot pressure distribution in elderly adults, few studies have been conducted to examine spatiotemporal gait characteristics, muscle activity and foot pressure distribution simultaneously. In addition, although the popularity of research into rehabilitation robotics has grown, there has been limited investigation of the impact of walking assist robots on foot pressure distribution in elderly adults. By using 3D motion capture, surface electromyography (sEMG) and foot pressure distribution at the same time, clinicians and researchers may gain a better understanding of alterations in spatiotemporal gait characteristics, muscle activity and foot pressure distribution during robot-assisted gait in elderly adults [14].

The purpose of this study was to investigate the effects of the new wearable hip-assist robot called the Gait-enhancing Mechatronic System (GEMS) (Samsung Electronics Co, Ltd., Suwon, Korea) on gait performance (spatiotemporal gait characteristics and muscle activity) and foot pressure distribution in elderly adults. In this study, we simultaneously analyzed spatiotemporal parameters, muscle activity and foot pressure distribution during walking with and without the GEMS in order to gain a comprehensive understanding of the role of the GEMS during gait in elderly adults. We hypothesized that when assisted by the GEMS, users would improve spatiotemporal gait parameters, reduce muscle activity and increase foot pressure compared to free walking and thereby walk more efficiently.

## Methods

### Participants

A total of 30 eligible subjects were recruited for the study and the characteristics of these subjects are shown in Table 1. Study inclusion criteria were: being medically stable, age between 65 and 84 years, no neurological or musculoskeletal abnormalities affecting gait and the ability to walk at least 10 m regardless of the use of assist devices [15]. Shoe size and foot imprints were taken to determine insole size. Subjects demonstrated high levels of physical performance, with a Short Physical Performance Battery (SPPB) score of 7 or higher (minimal-mild limitations) [16]. Exclusion criteria included a history of diseases that affect walking capacity, efficiency and endurance (e.g., lower extremity orthopedic diseases, neurologic disorders, cardiovascular disease, heart failure, or uncontrolled hypertension) and severe visual impairment or dizziness that could increase the risk of falls. Selection bias occurs at the stage of recruitment of participants; all subjects in this study had right leg dominance.

**Table 1:** Participant Characteristics

Characteristic	Value
Sex (male/female)	14/16
Age, years (mean ± SD)	74.10 ± 4.18
Height, cm (mean ± SD)	160.60 ± 7.68
Weight, kg (mean ± SD)	62.07 ± 9.08
Short Physical Performance Battery score, points (mean ± SD)	9.14 ± 1.23

*SD* Standard Deviation

### Study setting

The study was performed in the Neuroplasticity, Neurorehabilitation and Imaging (NEURI) Laboratory at Samsung Medical Center, Korea, and all subjects provided written informed consent before measurements. All procedures were approved by the ethics committee of the Samsung Medical Center Institutional Review Board.

### Wearable hip-assist robot

**Fig. 1:**
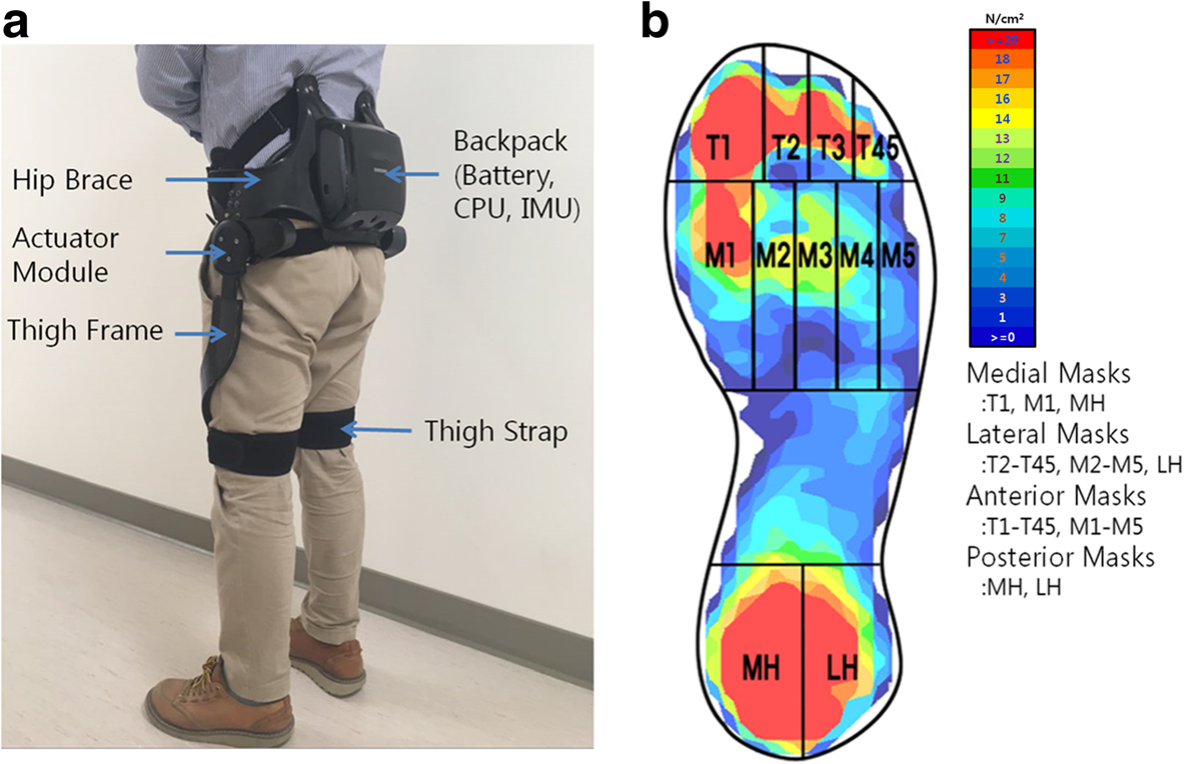
**a** Gait-enhancing Mechatronic System (GEMS). **b** Segmented foot regions on the insole. *T1* 1st toe, *T2* 2nd toe, *T3* 3rd toe, *T45* 4th–5th toes, *M1* 1st metatarsal, *M2* 2nd metatarsal, *M3* 3rd metatarsal, *M4* 4th metatarsal, *M5* 5th metatarsal, *MH* medial heel, *LH* lateral heel

The GEMS is a hip-type robotic exoskeleton developed by the Samsung Advanced Institute of Technology. The GEMS is worn around the waist and fastened at the waist and thighs by a set of belts with Velcro to assist motion directly at the hip joints (Fig. 1 a). The device weighs 2.9 kg and carries all of its electronics, computation, actuation, and power source (rechargeable lithium ion battery) in the backpack of the device. The normal operation time for the device is 1.3 h.

The device consists of a pair of actuators that generate assist torque to each hip joint. Assist torque is generated by two 70-W brushless DC motors that are mounted near the hip joints. The generated torque is transmitted to each joint through a 75:1 multi-stage gear system which can generate up to 14 Nm. Each joint has 1 active degree of freedom (DOF) for extension and flexion in the sagittal plane and 1 passive DOF for abduction and adduction in the frontal plane. A pair of special thigh frames transmits assist power from the actuators to the thighs while conforming to a user’s thigh contours [17]. The GEMS is available in two sizes to fit various waist/hip sizes: small (for hip circumferences 70~90 cm) and medium (for hip circumferences 90~100 cm). The difference between the two sizes is the size of the two side hip braces. The width of each version can be adjusted further to fit individual body size within the circumference range. Also the thigh frames are available in three sizes (large, medium, and small) for different leg lengths [18]. There are two types of sensors to measure the hip joint angles: potentiometers at the hip joints and encoders in the motors. Also there is an inertial measurement unit (IMU) in the backpack to measure rotation and acceleration of the device.

**Fig. 2:**
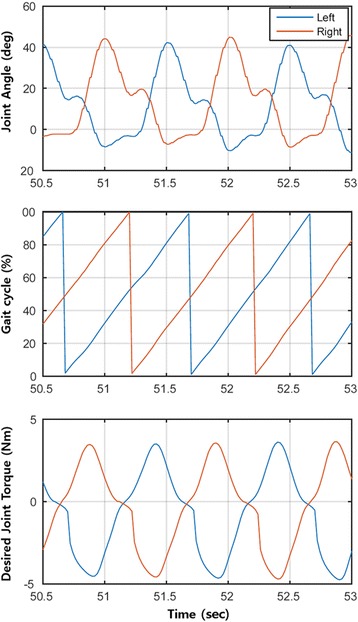
Device left and right joint angle, estimated gait cycle for each joint, and desired left and right joint torque. Positive value is the flexion

The gait assist controller consists of gait cycle estimation, speed estimation, and a joint torque generator which has been explained in detail by Seo et al. [19, 20]. In short, the controller takes input from two hip joint angle sensors to estimate the current gait cycle with respect to each leg using a particularly-shaped adaptive oscillator (PSAO) [19]. It then calculates the assistive torque by identifying the torque value associated with the estimated gait cycle from a predetermined torque pattern over a gait cycle (Fig. 2). The assist torque profiles (*τ*_ext, *bio*_, *τ*_*flex*, *bio*_) are initially adopted from a biomechanical study and then empirically modified to minimize discomfort [19, 20]. Then the actual extension and flexion torque delivered to the user (*τ*_*ext*_, *τ*_*flex*_) are the torque profiles (*τ*_ext, *bio*_, *τ*_*flex*, *bio*_) scaled by the walking speed scale factor (*α*_ext, *vel*_, *α*_*flex*, *vel*_), user body weight (*W*) and user preferred assist level (*L* = 14, 16, 18, *or* 20%):

$$ {\tau}_{ext}={\tau}_{\mathit{\operatorname{ext}}, bio}{\alpha}_{\mathit{\operatorname{ext}}, vel}W\ L $$

$$ {\tau}_{flex}={\tau}_{flex, bio}{\alpha}_{flex, vel}W\ L $$

where

$$ {\upalpha}_{\mathit{\operatorname{ext}}, vel}=\left\{\begin{array}{c}0.47,\kern0.5em if\ v\le 0.6m/s\\ {}\ 0.73,\kern0.5em if\ v\ge 1.3m/s\kern0.5em \\ {}0.2\ \left(v-0.6\right)+0.47,\kern1em if\ 0.6<v\le 1.0m/s\\ {}0.6\left(v-1\right)+0.55,\kern0.5em if\ 1.0<v\le 1.3m/s\end{array}\right. $$

$$ {\upalpha}_{flex, vel}=\left\{\begin{array}{c}0.34,\kern0.5em if\ v\le 0.6m/s\\ {}\ 0.70,\kern0.5em if\ v\ge 1.3m/s\kern0.5em \\ {}0.40\ \left(v-0.6\right)+0.34,\kern2em if\ 0.6<v\le 1.0m/s\\ {}0.67\left(v-1.0\right)+0.50,\kern0.5em if\ 1.0<v\le 1.3m/s\end{array}\right. $$

and the walking speed (*v*) is determined in real time using the acceleration sensor as discussed in [20]. Using the above rule, the flexion peak torque used in this study ranged from 3.13 to 9.70 Nm (the mean and standard deviation of the peak torque are 5.46 ± 1.72), and the extension peak torque ranged from 3.79 to 10.12 Nm (the mean and standard deviation of the peak torque are 6.04 ± 1.69).

A physical therapist operates the device through a custom built application on a hand held tablet. The application is used to turn assist torque on/off and change the assist level. The assist level is chosen based on a verbal feedback from the subjects prior to each trial. The tablet also displays real-time device information such as joint angle, velocity, and assistance torque values.

### Experimental protocol

The investigator explained the study protocol and tasks subjects were required to perform. The SPPB was administered prior to participation in the study to determine whether subjects met the inclusion criteria, and all participants were evaluated by a physical therapist.

Testing procedures used for sEMG were previously established [21] (Noraxon USA Inc., Scottsdale, AZ). The skin was prepared for sEMG collection by shaving and cleansing with alcohol to reduce skin impedance. Electrodes were then placed over the midline of each muscle belly, parallel to the muscle fiber orientation, and the maximal voluntary contraction (MVC) test was performed for the each muscle. The MVC test is the suggested method of normalizing by the Surface Electromyography for the Non-invasive Assessment of Muscles (SENIAM) and Kinesiology’s guidelines and is the most widely employed normalization method [22]. For the MVC test, subjects performed 5 s MVC against manual resistance from a physical therapist, with a 60s rest between each test [23]. MVC test positions for the selected muscles were determined based on descriptions in the muscle testing book [24]. In addition, footswitches (Model 500 DTS FootSwitch; Noraxon USA Inc., Scottsdale, AZ) were placed on the right toe and heel to identify the timing of stance and swing gait phases, and an F-scan system (Tekscan Inc., Boston, MA, USA) was placed in the subjects’ shoes in the form of insoles to measure foot pressure during gait. The trajectories of 19 markers were then placed on anatomical landmarks, including both anterior superior iliac spines (ASISs), the sacrum and the lower extremities. Spatiotemporal parameters, sEMG and foot pressure data were simultaneously collected during task performance. Prior to testing, participants walked along a 10-m walkway for five min to adjust to the robot-assisted gait.

The participants were asked to walk at a self-selected normal speed along a 10-m walkway under the following three conditions in random order using a table of random numbers: free gait without robot assistance (FG), a 10-m walk without wearing the exoskeleton in order to measure baseline spatiotemporal parameters, muscle activity and foot pressure distribution; robot-assisted gait with zero torque (RAG-Z), a 10-m walk wearing the exoskeleton, but the desired torque was set to zero to verify the effect of wearing the exoskeleton on spatiotemporal parameters, muscle activity and foot pressure distribution; robot-assisted gait (RAG), a 10-m walk while wearing the exoskeleton and using the assist torque.

### Measurements

Gait performance was analyzed using a 3D motion capture system with six optoelectronic cameras (Motion Analysis Corporation, Santa Rosa, CA, USA, sampling frequency 120 Hz). The trajectories of 19 markers placed on anatomical landmarks, using the Helen Hayes marker model [25], were collected. Movement data were automatically converted to 3D coordinates with motion capture software, EVaRT version 5.0 (Motion Analysis Corporation, Santa Rosa, CA, USA). Spatiotemporal parameters including gait speed, cadence, stride length, step width and single support time were calculated for each gait cycle using Ortho Track 6.5 software (Motion Analysis Corporation, Santa Rosa, CA, USA).

Muscle activity during gait performance was recorded using sEMG with bipolar surface electrodes (Ag/AgCl). The sEMG signals from four muscles on the right side including the rectus femoris (RF), biceps femoris (BF), tibialis anterior (TA) and medial gastrocnemius (MG) were collected. Muscle activity was recorded in accordance with the recommendations of the SENIAM project [26]. To reduce movement artifacts, a sampling frequency of 1000 Hz was used. Data was then passed through a 10–350 Hz sixth order Butterworth band-pass filter and full-wave rectified with Noraxon software (MyoResearch XP Master Edition). In addition, the root mean squared (RMS) values of the signal were calculated using a sliding 100 ms window for analysis. Subsequently, the data was passed through a sixth order Butterworth low-pass filter with a 6 Hz cutoff frequency to create a linear envelope and normalized to MVC data obtained prior to tasks [27, 28]. The average normalized sEMG activity was processed within the selected phases of the gait cycle using MATLAB software (MathWorks, Inc., Natick, MA, USA) [29]. In this study, we couldn’t define gait phases by detecting relevant gait events. We adopted fixed gait sub-phases according to Perry’s report [30], the stance and swing phases are subdivided into a number of sub-phases: initial contact (0% of the gait cycle), loading response (0–12%), midstance (12–31%), terminal stance (31–50%), pre-swing (50–62%), initial swing (62–75%), mid-swing (75–87%), and terminal swing (87–100%).

Foot pressure distribution was recorded at 50 Hz using the F-Scan system. As described in the Tekscan user manual (Tekscan Research Software User Manual Version 7.0 Rev. J, 2014), we performed step calibration, and plantar pressure was measured using the F-Scan sensor (Model #3000E), which is made up of 960 individual pressure sensing locations and a 0.02-mm-thick polyester sheet. Measured parameters included maximum force and peak pressure for the total foot, medial masks (medial heel, 1st metatarsal and 1st toe), lateral masks (lateral heel, 2nd-5th metatarsals and 2nd-5th toes), anterior masks (1st-5th toes and 1st-5th metatarsals) and posterior masks (medial heel and lateral heel) [9, 31] (Fig. 1 b).

### Statistical analyses

Statistical analysis was performed using SPSS ver. 18 for Window software (SPSS Inc., Chicago, IL, USA). A repeated measures ANOVA was used to compare spatiotemporal gait parameters, muscle activity and foot pressure distribution data among the three different conditions, and Tukey’s honestly significant difference (HSD) test was used for post hoc analysis. *p* < 0.05 was considered to be statistically significant.

## Results

### Spatiotemporal gait parameters

As shown in Table 2, there were significant differences in gait performance among the three conditions with respect to gait speed, cadence, stride length and single support time. Specifically, gait speed in the RAG condition was significantly faster than in the FG and RAG-Z conditions (*p* < 0.05), and cadence in the RAG condition was significantly higher than in the FG (*p* < 0.05) and RAG-Z conditions (*p* < 0.01). In addition, stride length in the RAG condition was significantly longer than in the FG and RAG-Z conditions (*p* < 0.05), and single support time in the RAG condition was significantly longer than in the FG (*p* < 0.01) and RAG-Z conditions (*p* < 0.05).

**Table 2:** Spatiotemporal Gait Parameters

	FG	RAG-Z	RAG
Gait Speed (cm/s)	97.94 ± 15.28	97.98 ± 15.52	110.71 ± 13.14^*†^
Cadence (step/min)	107.90 ± 5.80	105.48 ± 8.54	113.36 ± 6.92^*††^
Stride Length (cm)	107.23 ± 15.56	108.24 ± 13.57	117.76 ± 12.95^*†^
Step Width (cm)	11.64 ± 3.03	11.01 ± 2.94	11.85 ± 3.96
Single Support Time (%cycle)	36.02 ± 2.73	36.46 ± 2.81	38.64 ± 2.14^**†^

Values are expressed a mean ± standard deviation

*FG* free gait without robot assistance, *RAG-Z* robot-assisted gait with zero torque, *RAG* robot-assisted gait

^*^Different from the FG condition (*p* < 0.05), ^**^Different from the FG condition (*p* < 0.01)

^†^Different from the RAG-Z condition (*p* < 0.05), ^††^Different from the RAG-Z condition (*p* < 0.01)

### Muscle activity in walking with the GEMS throughout the gait cycle

**Fig. 3:**
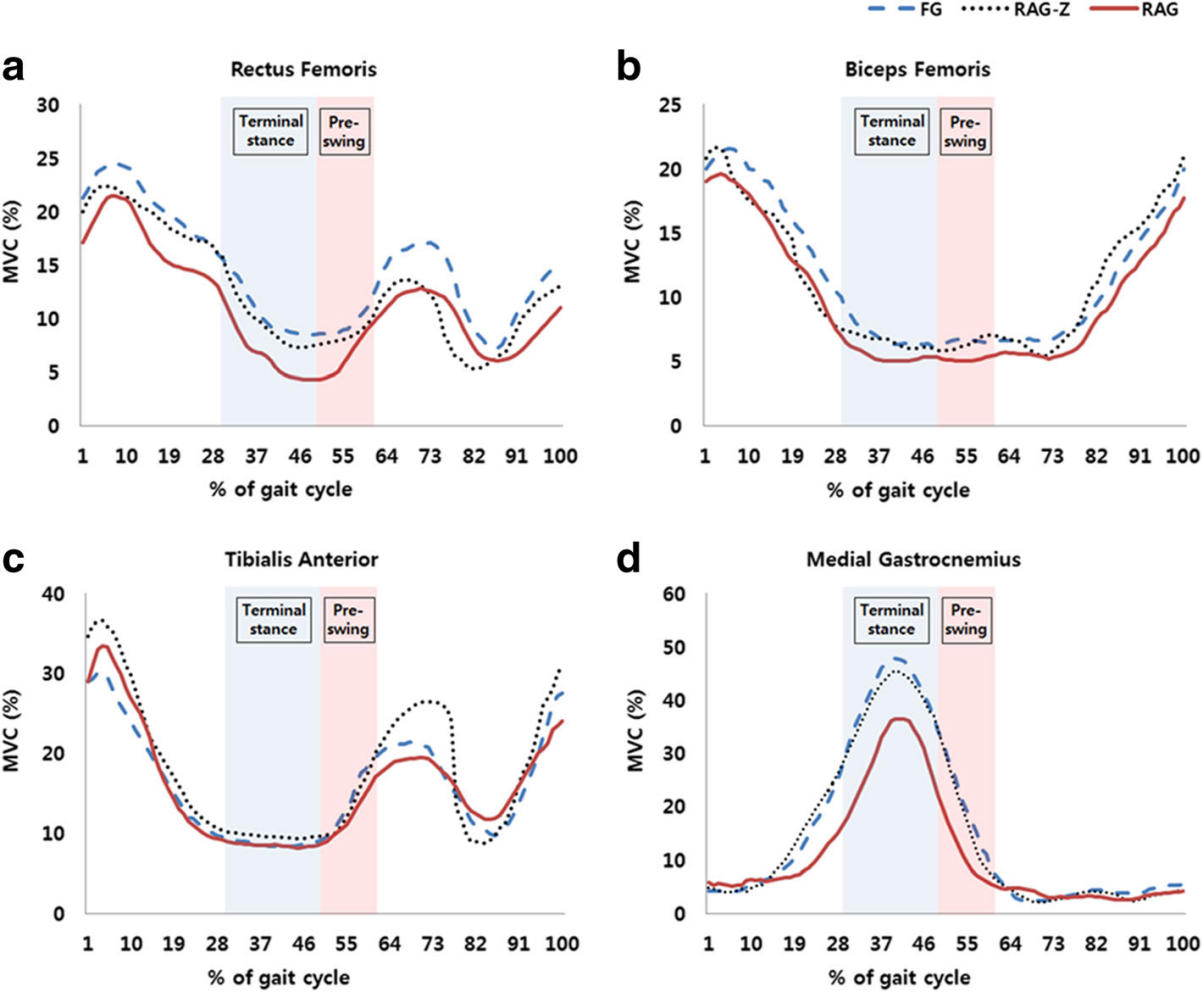
Average EMG activity (%MVC) of measured muscles during one gait cycle. **a** Average sEMG activity of rectus femoris during one gait cycle. **b** Average sEMG activity of biceps femoris during one gait cycle. **c** Average sEMG activity of tibialis anterior during one gait cycle. **d** Average sEMG activity of medial gastrocnemius during one gait cycle. *FG* free gait without robot assistance, *RAG-Z* robot-assisted gait with zero torque, *RAG* robot-assisted gait, *sEMG* surface electromyography, *MVC* maximum voluntary contraction

Figure 3 shows the average sEMG activity (%MVC) of measured muscles during one gait cycle, which is defined by the period from heel contact of one foot to subsequent heel contact of the same foot. RF and MG muscle activity in the RAG condition were significantly reduced compared to in the FG and RAG-Z conditions throughout the terminal stance phase (31–50% of the gait cycle) (*p* < 0.05), and MG muscle activity in the RAG condition was significantly reduced compared to in the FG and RAG-Z conditions throughout the pre-swing phase (50–62% of the gait cycle) (*p* < 0.05).

### Foot pressure distribution

Foot pressure distribution was significantly different during gait performance among the three conditions. As shown in Table 3, a significantly higher maximum force and peak pressure of the total foot were observed in the RAG condition compared with the FG (*p* < 0.01) and RAG-Z (*p* < 0.05) conditions. In the medial masks, maximum force and peak pressure were significantly higher in the RAG condition compared with the FG and RAG-Z conditions (*p* < 0.05). In the anterior masks, significantly higher maximum force was seen in the RAG condition compared to the FG and RAG-Z (*p* < 0.01), and significantly higher peak pressure was seen in the RAG condition compared to the FG and RAG-Z conditions (*p* < 0.05). In addition, in the posterior masks, a significantly higher maximum force was observed in the RAG condition compared to the FG and RAG-Z conditions (*p* < 0.05).

**Table 3:** Foot Pressure Distribution under Three Conditions (*N* = 30)

Masks	Parameters	FG	RAG-Z	RAG
Total Foot	MxF (%BW)	112.85 ± 10.30	119.56 ± 12.06	128.97 ± 14.55^**†^
	PP (N/cm^2^)	34.63 ± 11.32	35.72 ± 10.73	48.24 ± 18.49^**†^
Medial Masks	MxF (%BW)	20.92 ± 4.08	21.59 ± 4.33	24.21 ± 4.36^*†^
	PP (N/cm^2^)	22.28 ± 7.52	22.68 ± 7.67	30.69 ± 12.26^*†^
Lateral Masks	MxF (%BW)	13.75 ± 2.29	13.94 ± 2.89	13.83 ± 3.85
	PP (N/cm^2^)	18.63 ± 4.84	19.52 ± 6.05	17.06 ± 5.06
Anterior Masks	MxF (%BW)	10.89 ± 1.69	11.37 ± 1.75	13.48 ± 2.42^**††^
	PP (N/cm^2^)	16.47 ± 4.14	16.35 ± 3.63	20.01 ± 5.84^*†^
Posterior Masks	MxF (%BW)	31.66 ± 6.56	32.84 ± 5.76	37.81 ± 7.96^*†^
	PP (N/cm^2^)	4.30 ± 1.87	4.65 ± 1.76	5.29 ± 1.99

Values are expressed as number or mean ± standard deviation

Medial Masks: Medial heel, 1st metatarsal, 1st toe

Lateral Masks: Lateral heel, 2nd-5th metatarsals, 2nd-5th toes

Anterior Masks: 1st-5th toes, 1st-5th metatarsals

Posterior Masks: Medial heel, Lateral heel

*FG* free gait without robot assistance, *RAG-Z* robot-assisted gait with zero torque, *RAG* robot-assisted gait, *MxF (%BW)* maximum force (%body weight), *PP* peak pressure

^*^Different from the FG condition (*p* < 0.05), ^**^Different from the FG condition (*p* < 0.01)

^†^Different from the RAG-Z condition (*p* < 0.05), ^††^Different from the RAG-Z condition (*p* < 0.01)

## Discussion

The purpose of this study was to investigate the effects of the new wearable hip-assist robot, GEMS on spatiotemporal gait characteristics, muscle activity and foot pressure distribution in elderly adults. The present study demonstrated that the GEMS provided significant improvements including gait speed, cadence, stride length and single support time. Furthermore, the device reduced muscle activity of RF and MG throughout the terminal stance and muscle activity of MG throughout the pre-swing phases. In addition, the GEMS increased maximum force and peak pressure of the total foot, medial masks, anterior masks and posterior masks. These results support our hypothesis that the assistance provided by the GEMS would improve gait performance and help older adults to walk efficiently.

Gait speed may be used to identify elderly adults at increased risk of early mortality. Slower gait speed is associated with increased risk for falls, and each 10 cm/s decrease in gait speed was associated with a 17% increased risk of falls [32]. Gait speed is associated with survival among the elderly and reflects health and functional status. Gait speeds faster than 100 cm/s suggest healthier aging while gait speeds slower than 60 cm/s indicate a likelihood of poor health and function. According to the data from a cohorts study that evaluated the relationship between gait speed and survival [33], a gait speed faster than 100 cm/s suggests a better-than-average life expectancy and speeds above 120 cm/s denote exceptional life expectancy. One of the positive variables we quantified in this study was gait speed, which is associated with gait function in the elderly. The RAG condition increased gait speed to greater than 100 cm/s (110.71 cm/s), which is in line with a previous study on wearable robot-assisted gait in elderly adults [34]. A possible explanation for the effects of the GEMS on gait speed is that the device provides assistance at the hip joint, which pulls the leg upwards and forward during the swing phase, increasing cadence and stride length. Higher gait speed is achieved by increases in cadence and stride length [35, 36]. In terms of increasing gait speed, a different argument could be that forcing subjects to walk faster actually introduces fall and trip risks. However, according to the study by Quach et al. [37], slower gait (<60 cm/s) is related to an increased rate of indoor falls, whereas faster gait (≥130 cm/s) is related to an increased rate of outdoor falls. Also, normal gait speed (≥100 cm/s and <130 cm/s) showed the lowest rate of falls. In our study, gait speed in the RAG condition was 110.71 cm/s (normal gait speed/lower rate of falls). This result may indicate that increased gait speed with the assistance of GEMS was not too fast to increase the risk of falls. The results in this study indicate that disordered gait, defined as a gait that is slowed, is common with aging; however, elderly adults can improve their gait performance with the assistance of the GEMS.

In agreement with what was observed in other studies [7, 12], our results show that when assisted by the GEMS in the RAG condition, users reduced muscle activity compared to walking in the FG and RAG-Z conditions. Although the device provides assistance at the hip joint only, we found reduced muscle activity in both the hip flexor muscle (RF) and ankle plantar flexor muscle (MG). According to recent studies [7, 38, 39], there are at least three concomitant strategies that can be used by humans when walking: the ankle strategy, hip flexor strategy and hip extensor strategy. External assistance provided by a robotic exoskeleton could alter the joint trajectories [40, 41] and the physiological equilibrium by making one of the walking strategies more convenient than the others [12]. In our protocol, the hip assistance provided by the GEMS could modify normal physiological equilibrium by reducing the need for ankle push-off, thus lowering muscle activity of MG, as well as decreasing the use of the hip strategies and consequently lowering muscle activity of RF. All subjects in this study were healthy old adults who didn’t have any unilateral disease such as paralysis or arthritis. Therefore, we intended to collect data from multiple muscle sites of dominant leg (right side for all subjects) with an assumption that their gait pattern is symmetrical.

Beyond reduced hip flexor and ankle plantar flexor activity, we also observed a remarkable increase in maximum force and peak pressure of the total foot, medial masks, anterior masks and posterior masks in the RAG condition. Because foot pressure distribution is affected by gait speed and stride variability [9], the increase in maximum force and peak pressure in the total foot associated with the GEMS may be related to increased gait speed and stride length. In the medial masks, increased maximum force and peak pressure imply that elderly adults preferentially bear weight on the lateral foot during free walking [42]; however, in the RAG condition, maximum force and peak pressure improved significantly, so weight bearing on the medial foot compensated for the forces and heavy loads imposed on the foot. Walking may present a challenge to elderly adults with an age-related decline in foot pressure. Elderly adults show lower foot pressure and force in the anterior masks and have a reduced ability to push off in anticipation of the swing phase compared to young adults [43]. In the RAG condition, statistically significant changes in the anterior masks indicated that despite reduced ankle plantar flexor effort, greater force and pressure in the anterior masks increased the efficiency of walking. Foot pressure under the heel is affected by weight bearing at the heel strike. Elderly adults exert lower maximum force and pressure on the calcaneus region, resulting in instability of the ankle during the heel contact phase. However, in the posterior masks, an increase of maximum force was observed in the RAG condition. This indicated that the device provided the force needed to stabilize the ankle during the heel contact phase.

Walking places demands on multiple organ systems, including the musculoskeletal (bone, muscles and joints), cardiopulmonary (heart and lungs) and nervous systems (brain, spinal cord and peripheral nerves) [33, 44]. Slowing gait may reflect both multiple small changes in several different systems and a high energy cost of walking. [45]. In older adults, high energy cost of walking (e.g., inefficient) is a major factor in the age-related decline in activity and physical function [11]. Our previous study revealed that the GEMS made walking more efficient by decreasing metabolic energy used during walking in older adults [46]. To perform successful tasks, the highly skilled mover uses only the minimum muscle, joint motion and neural capacities necessary, and as a result movement is efficient because only the minimum capacities are selected [11]. Although we couldn’t assess joint motion and neural capacities, a clear reduction of hip flexor and ankle plantar flexor was observed. The data in this study showed reduced muscle activity in both hip flexor (RF) and ankle plantar flexor (MG) muscles and increased foot pressure. This result indicated that walking efficiency during push-off phases was increased by reduction of muscle activities required to walk and by increased foot pressure.

### Limitations

This study has a number of limitations. First, we could not evaluate the effects of the GEMS on the kinetics of walking. Thus, we could not assess the peak moment of the hip flexor, knee extensor and ankle plantar flexor, which contribute to push-off power. Further insights could be obtained in the future by recording kinematics and ground reaction forces simultaneously. Another limitation was that we only demonstrated the temporary effect of the device in this study. Further research should investigate the long-lasting effect of the GEMS on gait function as well as its effect on ADL in the elderly.

## Conclusions

The present study shows that the newly developed GEMS is useful for improvement of gait performance in older adults. The device enables elders to walk more efficiently by enhancing push-off power and walking speed and reducing muscle activity. In this study, we used the GEMS to provide assist torque to the hip joint only. Nonetheless, the data showed a reduction of both hip flexor and ankle plantar flexor activation and an increase in foot pressure in the terminal stance and pre-swing phases. This result suggests that the GEMS may be useful for several groups of people who have reduced hip or ankle plantar flexion torque. In the near future, we will apply this device to subjects showing different types of gait disorders and investigate long-term effects.

## Abbreviations

ADL: activities of daily living
BF: biceps femoris
FG: free gait without robot assistance
GEMS: Gait Enhancing Mechatronic System
MG: medial gastrocnemius
MxF: maximum force
PP: peak pressure
RAG: robot-assisted gait
RAG-Z: robot-assisted gait with zero torque
RF: rectus femoris
sEMG: surface electromyography
TA: tibialis anterior
